# MRI radiomics prediction modelling for pathological complete response to neoadjuvant chemoradiotherapy in locally advanced rectal cancer: a systematic review and meta-analysis

**DOI:** 10.1007/s00261-025-04953-5

**Published:** 2025-04-28

**Authors:** Jason Rai, Dinh V. C. Mai, Ioanna Drami, Edward T. Pring, Laura E. Gould, Phillip F. C. Lung, Thomas Glover, Joshua D. Shur, Brandon Whitcher, Thanos Athanasiou, John T. Jenkins

**Affiliations:** 1 BiCyCLE Research Group, St Mark’s the National Bowel Hospital, London, UK; 2 Department of Surgery and Cancer, Imperial College London, London, UK; 3 Department of Radiology, St Mark’s the National Bowel Hospital, London, UK; 4 Department of Radiology, The Royal Marsden NHS Foundation Trust, London, UK; 5 Research Centre for Optimal Health, University of Westminster, London, UK

**Keywords:** Magnetic resonance imaging, Radiomics, Rectal cancer, Neoadjuvant treatment, Pathological response

## Abstract

**Purpose:**

Predicting response to neoadjuvant therapy in locally advanced rectal cancer (LARC) is challenging. Organ preservation strategies can be offered to patients with complete clinical response. We aim to evaluate MRI-derived radiomics models in predicting complete pathological response (pCR).

**Methods:**

Search included MEDLINE, Embase and Cochrane Central Register of Controlled Trials (CENTRAL) and Cochrane Database of Systematic Reviews (CDSR) for studies published before 1st February 2024. The Quality Assessment of Diagnostic Accuracy Studies 2 (QUADAS-2) and Radiomics Quality Score (RQS) tools were used to assess quality of included study. The research protocol was registered in PROSPERO (CRD42024512865). We calculated pooled area under the receiver operating characteristic curve (AUC) using a random-effects model. To compare AUC between subgroups the Hanley & McNeil test was performed.

**Results:**

Forty-four eligible studies (12,714 patients) were identified for inclusion in the systematic review. We selected thirty-five studies including 10,543 patients for meta-analysis. The pooled AUC for MRI radiomics predicted pCR in LARC was 0.87 (95% CI 0.84–0.89). In the subgroup analysis 3 T MRI field intensity had higher pooled AUC 0.9 (95% CI 0.87–0.94) than 1.5 T pooled AUC 0.82 (95% CI 0.80–0.83) *p* < 0.001. Asian ethnicity had higher pooled AUC 0.9 (95% CI 0.87–0.93) than non-Asian pooled AUC 0.8 (95% CI 0.75–0.84) *p* < 0.001.

**Conclusion:**

We have demonstrated that 3 T MRI field intensity provides a superior predictive performance. The role of ethnicity on radiomics features needs to be explored in future studies. Further research in the field of MRI radiomics is important as accurate prediction for pCR can lead to organ preservation strategy in LARC.

**Graphical abstract:**

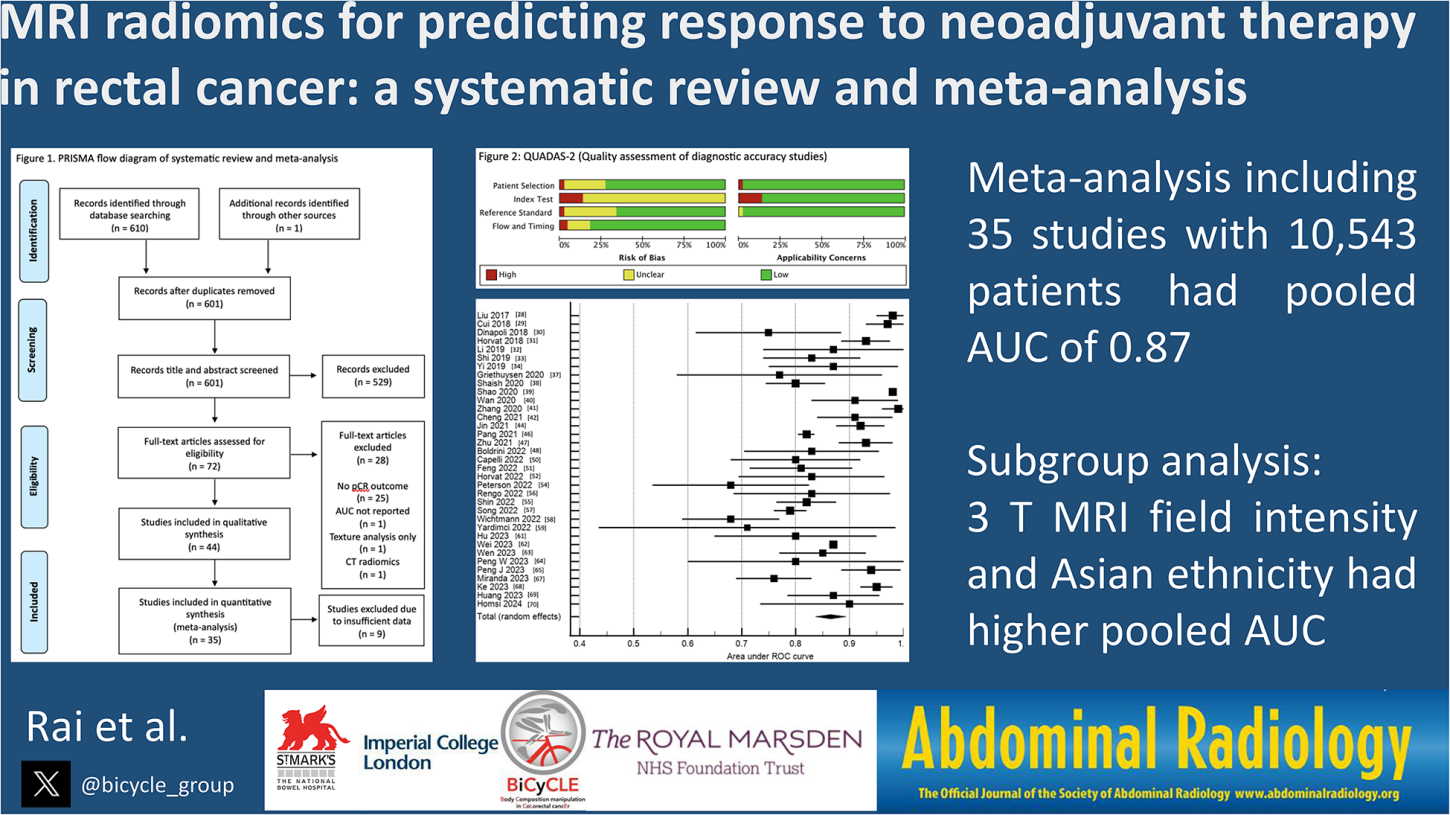

**Supplementary Information:**

The online version contains supplementary material available at 10.1007/s00261-025-04953-5.

## Background

Colorectal cancer (CRC) is the third commonest malignancy and the second leading cause of cancer mortality in the world [1]. Rectal cancer represents approximately one third of such CRC patients [1]. Treatment for locally advanced rectal cancer (LARC), defined as stage II (T3-4, node negative) or stage III (node positive) disease, has improved over the past few decades [2–5]. The current widely accepted standard of care for LARC consists of neoadjuvant chemoradiotherapy (nCRT) followed by total mesorectal excision (TME) and adjuvant chemotherapy [6–8]. This current approach has improved oncological outcomes but is associated with significant adverse effects such as gastrointestinal, urinary and sexual dysfunction [5, 9, 10].

Response to neoadjuvant therapy is assessed using qualitative methods based on clinical examination, endoluminal assessment and magnetic resonance imaging (MRI). Computed tomography, positron emission tomography and rectal ultrasound can be used in conjunction to assess response to nCRT but the most accurate method is MRI [7, 11]. Pathological complete response (pCR), defined as no evidence of residual viable tumour cells in the surgically resected specimen following nCRT, is reported in approximately 15–27% of LARC patients [12, 13]. If a patient achieves complete clinical response (cCR) to nCRT then a “watch-and-wait” strategy can be considered [14, 15].

Organ preservation has favourable functional outcome, good quality of life and it may lead to sphincter preservation and avoid the need for permanent stoma [16, 17]. Total neoadjuvant therapy (TNT) is an emerging strategy where both systemic chemotherapy and nCRT are delivered prior to surgery [18, 19]. TNT has been reported to further decrease the risk of distant metastases with improved disease-free survival (DFS), overall survival (OS) and higher rate of pCR [20]. However, TNT can lead to higher risk of toxicity [21]. The current challenge in LARC management is predicting patients that will benefit from neoadjuvant treatment strategy.

Prediction of treatment response in LARC is highly challenging due to the heterogenous nature of the disease. Radiomics is a rapidly developing field of research focused on the extraction of quantitative features from medical imaging. Radiomics refers to the characterisation of tumour phenotypes based on a diverse array of quantitative radiomic features including shape, intensity, texture-based and higher-order features [22, 23]. Research and application of radiomics in the field of LARC can be opportunistic as patients routinely undergo MRI of the pelvis for local disease staging. The radiomics workflow involves multiple steps with challenges including standardisation and adherence to machine learning best practice. Therefore, there is a need to assess the methodology of existing studies prior to considering its application in routine clinical practice. We aim to summarise the current literature on the performance of MRI radiomics prediction models for identifying pCR in LARC, evaluate the quality of methodology and assess for risk of bias.

## Methods

This systematic review and meta-analysis is reported according to the “Preferred Reporting Items for Systematic reviews and Meta-Analyses” (PRISMA) statement. The research protocol was registered in PROSPERO (CRD42024512865).

### Search strategy

MEDLINE, Embase and Cochrane Central Register of Controlled Trials (CENTRAL) and Cochrane Database of Systematic Reviews (CDSR) searches were conducted by an information specialist. The schematic search was as follows: rectal cancer, rectal neoplasms, magnetic resonance imaging, radiomics, artificial intelligence and machine learning. See supplementary Table 1 for detailed search strategies. The search was conducted until 1st February 2024.

### Eligibility criteria

Studies investigating the application of radiomics model for predicting response to neoadjuvant therapy were selected using the following criteria: locally advanced rectal cancer patients without metastases (cT3-4, N0, M0 or any cT, N1-2, M0), adenocarcinoma, neoadjuvant therapy and outcome defined as pCR. The following studies were excluded: studies that included patients with immunotherapy as neoadjuvant therapy, neuroendocrine tumours, outcome defined as tumour regression without pCR, non-English studies, conference abstracts and systematic reviews.

### Article selection and data extraction

Following the eligibility criteria two independent reviewers (JR and DM) screened titles and abstracts using Covidence. The same independent reviewers performed subsequent full text eligibility assessment. Reasons for the exclusion of articles were recorded. Inclusion of articles had been approved by both reviewers. Disagreements between the two reviewers were resolved by discussion with third reviewer (ID). All three reviewers were medical doctors with over ten years of research experience. Supplementary documents from studies were explored for available data but authors were not contacted in case of missing data.

### Quality assessment

The Quality Assessment of Diagnostic Accuracy Studies (QUADAS-2) tool and Radiomics Quality Score (RQS) were used to assess methodological quality of the included studies [24, 25]. Two reviewers (JR and DM) separately analysed individual studies using QUADAS-2 and RQS. Disagreements between the two reviewers were resolved by discussion with third reviewer (ID). Publication bias was assessed using the Egger regression test.

### Data synthesis

Predictive performance of a model is frequently quantified and reported using the receiver operating characteristic (ROC) curve and measure of area under the curve (AUC). If a study reported more than one radiomics prediction model, then the result of the model with the highest AUC was selected. The AUC of internal validation, or external validation where reported, was selected rather than the AUC of the training model. AUC of 1.0 indicates perfect discrimination. AUC values can be considered as poor (0.6–0.7), fair (0.7–0.8), good (0.8–0.9) and excellent (0.9-1.0) [26].

### Statistical analysis

Meta-analysis was performed using reported AUC with 95% confidence interval (CI). MedCalc version 22.016 (MedCalc Software Ltd, Ostend, Belgium) was used to pool AUC by calculating the standard error from 95% CI as reported by Zhou et al. under the random effects model [25]. To assess heterogeneity between studies, the inconsistency index (I^2^) was used. Heterogeneity was quantified as low, moderate and high using upper limits of 25, 50 and 75% for I^2^ respectively. A forest plot was drawn to show AUC in each study in relation to the summary pooled estimate.

## Results

### Search

**Fig. 1:**
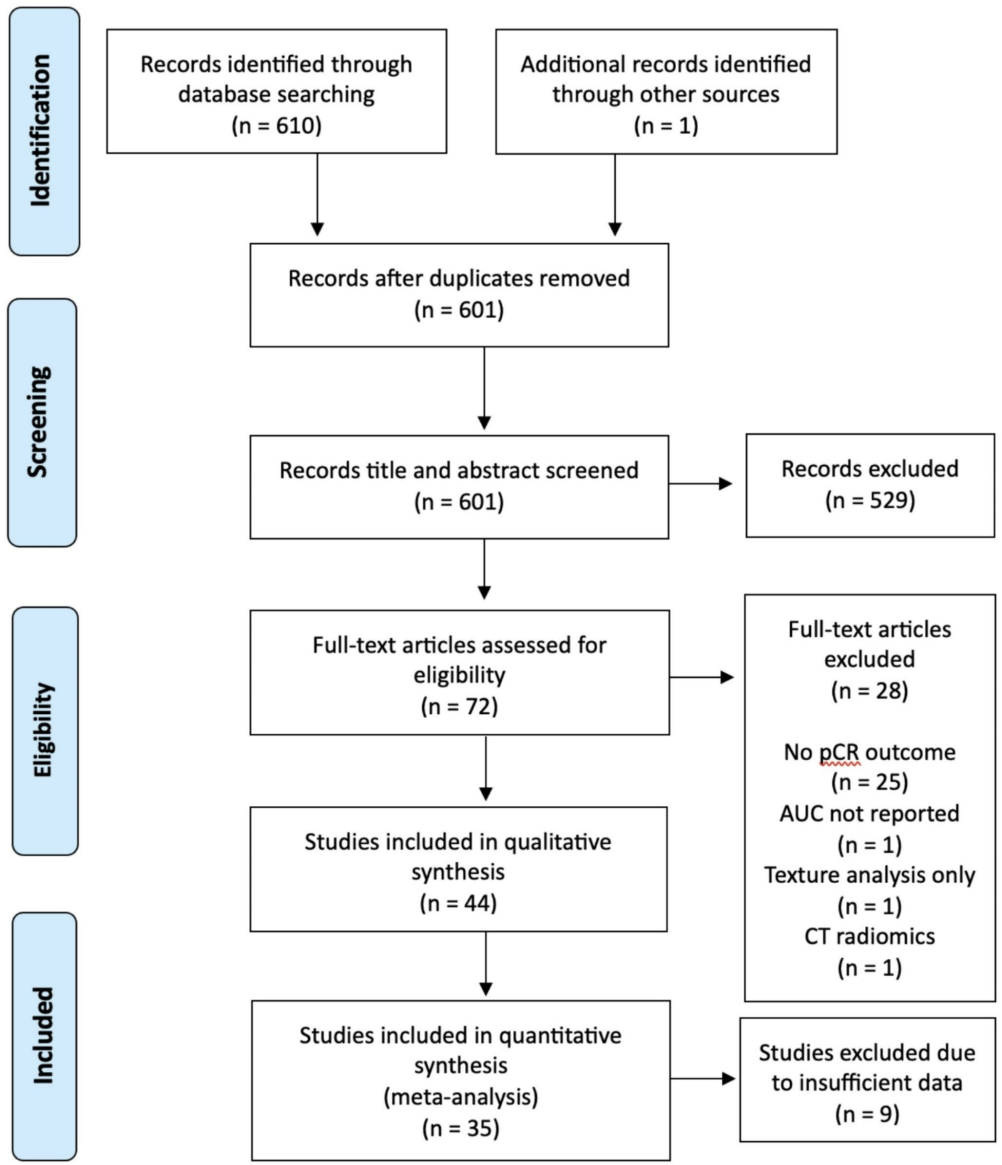
PRISMA flow diagram of systematic review and meta-analysis

A total of 610 studies were identified by the literature search. One study was identified through additional record using manual search as the authors were previously aware of this publication. This study was selected as it met the eligibility criteria. Following removal of duplicates, 601 titles and abstracts were screened for eligibility. In total 529 titles and abstracts were excluded, and 72 studies were assessed for full text review. Forty-four studies were included for data extraction and thirty-five studies were selected for the meta-analysis. See Fig. 1 the PRISMA flow diagram for an overview of the search process.

### Study population

**Fig. 2:**
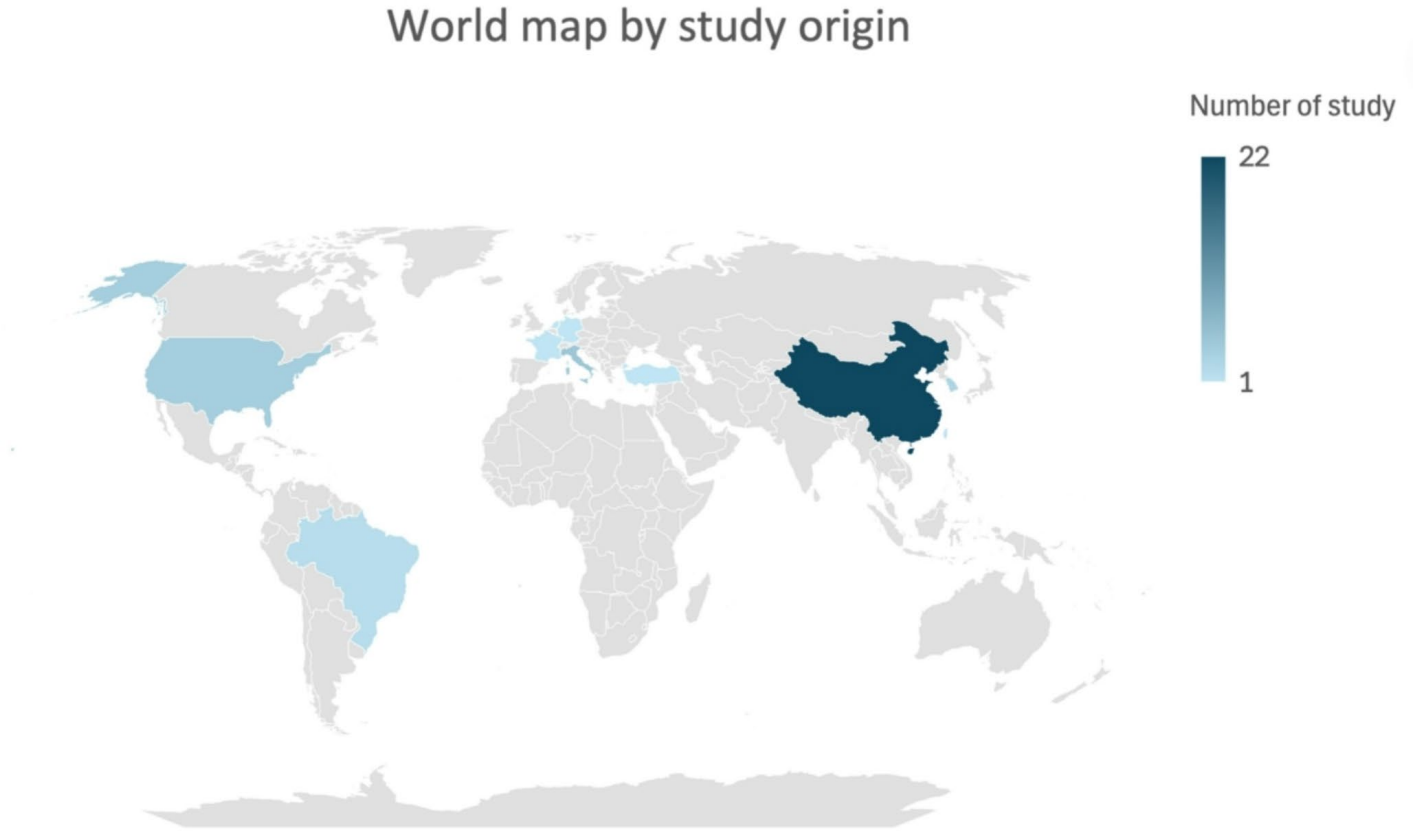
World heat map based on MRI derived radiomics study

Table 1 shows the LARC patient cohort treated between 2016 and 2024. The sample size from each study ranged from 48 to 1201. Figure 2 displays the distribution and concentration of studies in a world map. Twenty-six studies (59%) included Asians, nine studies (20%) included Europeans, four studies (9%) included north Americans, two studies included south Americans and three studies did not specify study populations. Twenty-three studies (52%) were single centre and twenty-one (48%) were multicentre. Five (11%) out of forty-four studies were reported as prospective.

**Table 1:** Study characteristics of MRI-derived radiomics model

No	Year	Author	Population	Study design	*N*	Centres	MRI Timing	MRI field intensity	Sequence	Neoadjuvant Radiotherapy (Cumulative)	Neoadjuvant Chemotherapy
1	2016	Nie et al. [27]	NR	R	48	1	Pre-nCRT	3 T	T2WI, T1WI	50 Gy	Capecitabine 825 mg/m2/BD Oxaliplatin 100 mg/m2
2	2017	Liu et al. [28]	China	R	222	1	Pre-nCRT Post-nCRT	3 T	T2WI, DWI	41.8–50.6 Gy	Capecitabine 825 mg/m2/BD
3	2018	Cui et al. [29]	China	R	186	1	Pre-nCRT	3 T	T2WI, cT1WI, ADC	50 Gy	Capecitabine 800 mg/m2/day
4	2018	Dinapoli et al. [30]	Italy	R	221	3	Pre-nCRT	1.5 T	T2WI	43.8–50.4 Gy	Capecitabine; capecitabin + oxaliplatin; flurouracil; oxaliplatin + flurouracil
5	2018	Horvat et al. [31]	Brazil	R	114	1	Pre-nCRT	1.5 T 3 T	T2WI, DWI	NR	NR
6	2019	Li et al. [32]	China	R	131	1	Pre-nCRT Post-nCRT	1.5 T	T2WI,	46–50 Gy	Capecitabine; 5-flurouracil; mFOLFOX; CAPOX
7	2019	Shi et al. [33]	NR	R	51	1	Pre-nCRT Mid-nCRT	3 T	T2WI, DWI, cT1WI, ADC	50 Gy	Capecitabine 825 mg/m2/BD for 5 weeks and Oxaliplatin 110 mg/m2/once every 3 weeks. On 5th week 5-flurouracil and oxaliplatin or capecitabine and oxaliplatin
8	2019	Yi et al. [34]	China	R	134	1	Pre-nCRT	1.5 T 3 T	T2WI	46–50 Gy	Capecitabine 825 mg/m2/day
9	2020	Antunes et al. [35]	USA	R	104	3	Pre-nCRT	1.5 T 3 T	T2WI	45–50.4 Gy	Capecitabine 825 to 850 mg/m2/day
10	2020	Cusumano et al. [36]	NR	R	195	2	Pre-nCRT	1.5 T 3 T	T2WI	50–55 Gy	Capecitabine or 5-flurouracil or capecitabine plus oxaliplatin
11	2020	Griethuysen et al. [37]	Netherlands	R	133	2	Pre-nCRT	1.5 T	T2WI, DWI, ADC	50.4 Gy	Capecitabine 825 mg/m2
12	2020	Shaish et al. [38]	Italy	R	132	2	Pre-nCRT	NR	T2WI	5 Gy	Capecitabine, 5-flurouracil, FOLFOX
13	2020	Shao et al. [39]	China	R	981	4	Pre-nCRT	1.5 T 3 T	T2WI, DWI	NR	NR
14	2020	Wan et al. [40]	China	R	165	1	Pre-nCRT	3 T	T2WI, DWI	25 Gy, 45–50.4 Gy	Capecitabine 825 mg/m2/BD; Capecitabine 1 g/m2/BD, Oxaliplatin 130 mg/m2
15	2020	Zhang et al. [41]	China	P	383	1	Pre-nCRT Post-nCRT	3 T	T2WI, DWI	NR	Capecitabine 825 mg/m2/day
16	2021	Cheng et al. [42]	China	R	193	1	Pre-nCRT	3 T	T2WI, T2FS, T1WI	45–50.4 Gy	mFOLFOX6 and CapeOX
17	2021	Jang et al. [43]	Korea	R	466	1	Pre-nCRT	1.5 T 3 T	T1WI, T2WI	50.4 Gy	5-FU, Capecitabine
18	2021	Jin et al. [44]	China	R + P	622	2	Pre-nCRT Post-nCRT	1.5 T 3 T	T2WI, DWI, T1WI, cT1WI	NR	NR
19	2021	Lee et al. [45]	Korea	R	912	1	Pre-nCRT	1.5 T 3 T	T2WI, cT1WI, DWI	NR	NR
20	2021	Pang et al. [46]	China	R	275	2	Post-nCRT	1.5 T	T2WI	45 Gy	5-flurouracil
21	2021	Zhu et al. [47]	China	R	472	1	Pre-nCRT	3 T	DWI	41.8–50.6 Gy	Capecitabine 825 mg/m2/day
22	2022	Boldrini et al. [48]	China	R	221	4	Pre-nCRT	1.5 T 3 T	T2WI	45 Gy	Oral Capecitabine 1650 mg/m2/day (d1-7,q7); 5-fluorouracil 225 mg/m2/day (d1-7,q7) OR iv Oxaliplatin 60 mg/m2 (d1,q7) plus oral capecitabin 1300 mg/m2/day (d1-7,q7)
23	2022	Bordron et al. [49]	France	R	124	2	Pre-nCRT	1 T 1.5 T 3 T	T2WI, DWI, CT	NR	NR
24	2022	Capelli et al. [50]	Italy	R	50	1	Pre-nCRT	3 T	T2WI, ADC, PET	NR	NR
25	2022	Feng et al. [51]	China	R + P	1033	5	Pre-nCRT	1.5 T 3 T	T2WI, DWI, cT1WI	50 Gy, 45 Gy	5-flurouracil based regimen combined with or without oxaliplatin
26	2022	Horvat et al. [52]	USA	R	164	2	Post-nCRT	1.5 T 3 T	T2WI, DWI	NR	NR
27	2022	Nardone et al. [53]	Italy	R	100	3	Pre-nCRT Post-nCRT	1.5 T	T2WI, DWI, ADC	45 Gy	Capecitabine 825 mg/m2/day
28	2022	Peterson et al. [54]	USA	R	131	1	Pre-nCRT	NR	T2WI	NR	NR
29	2022	Shin et al. [55]	Korea	R	898	1	Post-nCRT	1.5 T 3 T	T2WI, ADC	NR	NR
30	2022	Rengo et al. [56]	Italy	R + P	95	2	Pre-nCRT	1.5 T 3 T	T2W1	50.4–54 Gy	Oxaliplatin, 5 FU
31	2022	Song et al. [57]	China	R	674	4	Pre-nCRT	3 T	T2WI	Short course and long course	NR
32	2022	Wichtmann et al. [58]	Germany	R	129	5	Pre-nCRT Post-nCRT	1.5 T 3 T	T2WI, DWI, ADC	CAOAROAIO-12 study protocol	CAOAROAIO-12 study protocol
33	2022	Yardimci et al. [59]	Turkey	R	67	1	Pre-nCRT	1.5 T	T2WI	45–50.4 Gy	Capecitabine 825 mg/m2/BD
34	2023	Chou et al. [60]	Taiwan	R	133	1	Pre-nCRT	1.5 T	T2WI	45–54 Gy	Uracil-tegafur 200 mg/m2/d and mitocycin C 6 mg/m2
35	2023	Hu et al. [61]	China	P	99	15*	Pre-nCRT	NR	T2WI	46–50.4 Gy	FORWARC study protocol
36	2023	Wei et al. [62]	China	R	151	2	Pre-nCRT Post-nCRT	3 T	T2WI, DWI	50.4 Gy	XELOX; capecitabine; mFOLFOX6; 5-flurouracil and leucovorin
37	2023	Wen et al. [63]	China	R	126	1	Pre-nCRT Post-nCRT	1.5 T 3 T	T2WI	45–50 Gy	Capecitabine, XELOX, mFOLFOX6
38	2023	Peng W. et al. [64]	China	R	165	1	Pre-nCRT Post-nCRT	3 T	T2WI, DWI	45–50.4 Gy, 25 Gy	Capecitabine, Oxaliplatin
39	2023	Peng J. et al. [65]	China	R	83	1	Pre-nCRT Post-nCRT	3 T	T2WI, T1WI, cT1WI, DWI	50 Gy	Capecitabine 825 mg/m2/day, Oxaliplatin 50 mg/m2
40	2023	Ouyang et al. [66]	China	R	89	1	Pre-nCRT	3 T	T2WI	50.4 Gy	CAPOX
41	2023	Miranda et al. [67]	Brazil	R	180	1	Pre-nCRT Post-nCRT	1.5 T	T2WI	50.4 Gy	5-flurouracil, leucovorin
42	2023	Ke et al. [68]	China	R	1201	4	Pre-nCRT Post-nCRT	1.5 T 3 T	T2WI T1WI cT1WI DWI	46–50.4 Gy	5-FU, Oxaliplatin, mFOLFOX6 or FOLFOXIRI
43	2023	Huang et al. [69]	China	R	563	2	Pre-nCRT Post-nCRT	3 T	T2WI DWI cT1WI	45–50 Gy	5-Flurouracil
44	2024	Homsi et al. [70]	USA	R	98	2	Pre-nCRT	1.5 T 3 T	T2WI T1WI cT1WI DWI	25 and 40 Gy	Capecitabine, FOLFOX

(NR: Not reported. *FORWARC Trial.)

**Table 2:** Summary of MRI-derived radiomic model characteristics

No	Year	Author	VOI Software	Segmentation	Feature extraction software	Imaging features	Number of features	Classifier	Internal validation	External Validation
1	2016	Nie et al. [27]	MIM Maestro	Manual	NR	Haralick’s GLCM, time-intensity curves, wash-in slope, washout slope, histogram, signal intensity	103	DL	Yes	No
2	2017	Liu et al. [28]	ITK-SNAP	Manual	MATLAB	Statistical, Voxel-intensity, Wavelet	2252	SVM	Yes	No
3	2018	Cui et al. [29]	ITK-SNAP	Manual	AK	Histogram, texture, factor features	1188	LR	Yes	No
4	2018	Dinapoli et al. [30]	Eclipse	Manual	Moddicom	GTV volume, GTV surface, Histogram (skewness, kurtosis and entropy)	1200	LR	No	Yes
5	2018	Horvat et al. [31]	ITK-SNAP	Manual	MATLAB	Texture	34	RF	No	No
6	2019	Li et al. [32]	NR	Manual	IBEX	Tumour volume, intensity and texture	165	LR	Yes	No
7	2019	Shi et al. [33]	MIM Maestro	Manual	MATLAB	Textural, histogram	96	DL	Yes	No
8	2019	Yi et al. [34]	MaZda	Manual	MaZda	Gray level histogram, gradient, run-length matrix, co-occurrence matrix, autoregressive model and wavelet	340	SVM	Yes	No
9	2020	Antunes et al. [35]	3D Slicer	Manual	MATLAB	Texture	764	RF	Yes	Yes
10	2020	Cusumano et al. [36]	Eclipse	Manual	Moddicom	Fractal, statistical, textural and morphological	90	LR	Yes	No
11	2020	Griethuysen et al. [37]	MANGO	Manual and Semi-automatic	PyRadiomics	First order statistics, shape based, GLCM, GLRM, GLSZM, GLDM, NGTDM	2505	LR	No	Yes
12	2020	Shaish et al. [38]	3D Slicer	Manual	PyRadiomics	First order statistics, shape based, GLCM, GLRM, GLSZM, GLDM, NGTDM	3190	LR	Yes	No
13	2020	Shao et al. [39]	ITK-SNAP	Manual	PyRadiomics	First order statistics, shape based, GLCM, GLRM, GLSZM, GLDM, NGTDM	702	XGBoost	No	Yes
14	2020	Wan et al. [40]	Radcloud	Manual	Radcloud	Shape characteristic; first order statistic; texture feature: GLCM, GLDM, GLRM, GLSZM, NGTDM; high order statistical characteristics	1049	LR	Yes	Yes
15	2020	Zhang et al. [41]	ITK-SNAP	Manual	Python	N/A	N/A	DL	Yes	No
16	2021	Cheng et al. [42]	ITK-SNAP	Manual	PyRadiomics	First order statistics, shape based, GLCM, GLRM, GLSZM, GLDM, NGTDM	5901	LR	Yes	No
17	2021	Jang et al. [43]	N/A	N/A	MATLAB	N/A	N/A	DL	Yes	No
18	2021	Jin et al. [44]	NR	Automatic	N/A	N/A	N/A	DL	Yes	Yes
19	2021	Lee et al. [45]	3D slicer	Semi-automatic	PyRadiomics	First order statistics, shape based, GLCM, GLRM, GLSZM, GLDM, NGTDM	3740	RF	Yes	No
20	2021	Pang et al. [46]	U-Net	Automatic	PyRadiomics	N/A	N/A	SVM	Yes	Yes
21	2021	Zhu et al. [47]	ITK-SNAP	Manual	Python	N/A	N/A	DL	Yes	No
22	2022	Boldrini et al. [48]	NR	Manual	MODDICOM	Skewness, Entropy	NR	LR	Yes	Yes
23	2022	Bordron et al. [49]	3D Slicer	Manual	Miras	Shape, intensity, texture	822	DL	No	Yes
24	2022	Capelli et al. [50]	PMOD	Manual	PMOD	Histogram, gray-level cooccurrence matrix, run length matrix	34	LR	No	No
25	2022	Feng et al. [51]	ITK-SNAP	Manual	PyRadiomics	Texture, First-order, Wavelets	2106	SVM	Yes	Yes
26	2022	Horvat et al. [52]	ITK-SNAP	Manual	NR	Texture, Haralick, Gabor edges	91	RF	Yes	Yes
27	2022	Nardone et al. [53]	NR	Manual	LifeX	Texture	NR	LR	Yes	Yes
28	2022	Peterson et al. [54]	XNAT	Manual	PyRadiomics	First order statistics, shape based, GLCM, GLRM, GLSZM, GLDM, NGTDM	96	LR	Yes	No
29	2022	Shin et al. [55]	N/R	N/R	WEKA	N/R	N/R	Decision Trees J48	No	Yes
30	2022	Rengo et al. [56]	3D Slicer	Semiautomatic	PyRadiomics	NR	1132	LR	Yes	No
31	2022	Song et al. [57]	NR	Manual	Precision medicine open platform	Texture, statistical, wavelet, histograms	789	SVM	Yes	No
32	2022	Wichtmann et al. [58]	ITK-SNAP	Manual	DL	N/A	N/A	DL	Yes	Yes
33	2022	Yardimci et al. [59]	3D slicer	Manual	PyRadiomics	First order statistics, shape based, GLCM, GLRM, GLSZM, GLDM, NGTDM	1046	RF	Yes	No
34	2023	Chou et al. [60]	ITK-SNAP	Manual	PyRadiomics	First order statistics, shape based, GLCM, GLRM, GLSZM, GLDM, NGTDM	1223	LASSO	Yes	No
35	2023	Hu et al. [61]	ITK-SNAP	Automatic	DeepInteg	N/A	N/A	DL	Yes	No
36	2023	Wei et al. [62]	ITK-SNAP	Manual	PyRadiomics	First order statistics, shape based, GLCM, GLRM, GLSZM, GLDM, Wavelet, LoG	4528	RF	Yes	Yes
37	2023	Wen et al. [63]	MaZda	Manual	MaZda	Histogram, absolute gradient, run-length matrix, GLCM, AR model	250	LR	Yes	No
38	2023	Peng W. et al. [64]	Radcloud	Manual	PyRadiomics	Shape, First-order, Texture features	5636	LR	Yes	No
39	2023	Peng J. et al. [65]	ITK-SNAP	Manual	PyRadiomics	First order, GLRM, GLCM, NGTDM, GLSZM, GLDM	3720	KNN	Yes	No
40	2023	Ouyang et al. [66]	ITK-SNAP	Manual	PyRadiomics	First order statistics, shape based, GLCM, GLRM, GLSZM, GLDM, NGTDM	100	LR	Yes	No
41	2023	Miranda et al. [67]	ITK-SNAP	Manual	PyRadiomics	First order statistics, shape based, GLCM, GLRM, GLSZM, GLDM, NGTDM	2313	LASSO	Yes	No
42	2023	Ke et al. [68]	ITK-SNAP	Manual	DeepRP-RC	N/A	NR	DL	Yes	Yes
43	2023	Huang et al. [69]	DARWIN	Manual	PyRadiomics	First order statistics, shape based, GLCM, GLRM, GLSZM, GLDM, NGTDM	NR	SVM	Yes	Yes
44	2024	Homsi et al. [70]	Olea Sphere	Manual	NR	First order statistics, shape based, GLCM, GLRM, GLSZM, GLDM, NGTDM	540	DL	Yes	Yes

(NR: Not recorded. N/A: Not applicable. SVM: Support Vector Machine. LR: Logistic regression. RF: Random forest. DL: Deep learning. LASSO: Least Absolute Shrinkage and Selection Operator.)

**Table 3:** Summary of performance of MRI-derived radiomics model

No	Year	Author	TP	FP	FN	TN	Sensitivity	Specificity	PPV	NPV	AUC	AUC 95% CI	SE*
1	2016	Nie et al. [27]	-	-	-	-	-	-	-	-	0.84	-	-
2	2017	Liu et al. [28]	9	1	3	55	0.75	0.98	0.9	0.95	0.98	0.942–1	0.015
3	2018	Cui et al. [29]	-	-	-	-	0.96	0.89	-	-	0.97	0.924-1	0.020
4	2018	Dinapoli et al. [30]	-	-	-	-	-	-	-	-	0.75	0.61–0.88	0.069
5	2018	Horvat et al. [31]	-	-	-	-	1	0.91	0.72	1	0.93	0.87–0.96	0.023
6	2019	Li et al. [32]	6	3	3	32	0.67	0.91	0.67	0.91	0.87	0.74–1	0.066
7	2019	Shi et al. [33]	-	-	-	-	-	-	-	-	0.83	0.71–0.89	0.046
8	2019	Yi et al. [34]	-	-	-	-	0.81	0.90	-	-	0.87	0.75–0.99	0.061
9	2020	Antunes et al. [35]	7	10	3	24	0.70	0.71	0.41	0.89	0.71	-	-
10	2020	Cusumano et al. [36]	-	-	-	-	-	-	-	-	0.72	-	-
11	2020	Griethuysen et al. [37]	-	-	-	-	-	-	-	-	0.77	0.58–0.96	0.097
12	2020	Shaish et al. [38]	-	-	-	-	-	-	-	-	0.80	0.74–0.85	0.028
13	2020	Shao et al. [39]	-	-	-	-	0.96	0.98	0.94	0.99	0.98	0.98–0.99	0.003
14	2020	Wan et al. [40]	-	-	-	-	1	0.78			0.91	0.83–0.99	0.041
15	2020	Zhang et al. [41]	18	2	0	73	1	0.97	0.9	1	0.99	0.94-1	0.015
16	2021	Cheng et al. [42]	-	-	-	-	1	0.82	-	-	0.91	0.84–0.98	0.036
17	2021	Jang et al. [43]	6	3	14	90	0.3	0.96	0.67	0.87	0.76	-	-
18	2021	Jin et al. [44]	-	-	-	-	0.91	0.92	0.83	0.96	0.92	0.87–0.96	0.023
19	2021	Lee et al. [45]	-	-	-	-	0.72	0.60	-	-	0.84	-	-
20	2021	Pang et al. [46]	3	2	3	26	0.5	0.93	0.60	0.90	0.82	0.80–0.83	0.008
21	2021	Zhu et al. [47]	-	-	-	-	0.84	0.93	0.77	0.95	0.93	0.88–0.98	0.026
22	2022	Boldrini et al. [48]	-	-	-	-	0.7	0.64	0.28	0.91	0.83	0.71–0.96	0.064
23	2022	Bordron et al. [49]	4	5	1	50	0.8	0.91	0.44	0.98	0.81	-	-
24	2022	Capelli et al. [50]	13	14	1	22	0.93	0.61	0.48	0.96	0.8	0.68–0.92	0.061
25	2022	Feng et al. [51]	-	-	-	-	0.89	0.74	0.51	0.93	0.81	0.72–0.91	0.048
26	2022	Horvat et al. [52]	3	4	5	38	0.38	0.9	0.43	0.88	0.83	0.70–0.97	0.069
27	2022	Nardone et al. [53]	6	9	0	18	1	0.67	0.40	1	0.92	-	-
28	2022	Peterson et al. [54]	-	-	-	-	0.2	0.86	-	-	0.68	0.28–0.57	0.074
29	2022	Shin et al. [55]	-	-	-	-	0.86	1	1	0.94	0.83	0.65–0.94	0.074
30	2022	Rengo et al. [56]	57	66	18	165	0.76	0.71	0.46	0.90	0.82	0.77–0.88	0.028
31	2022	Song et al. [57]	-	-	-	-	-	-	0.94	0.91	0.79	0.77–0.83	0.015
32	2022	Wichtmann et al. [58]	-	-	-	-	-	-	-	-	0.68	0.59–0.77	0.046
33	2022	Yardimci et al. [59]	-	-	-	-	0.81	0.57	0.81	0.57	0.71	0.41–0.96	0.140
34	2023	Chou et al. [60]	-	-	-	-	-	-	-	-	0.86	-	-
35	2023	Hu et al. [61]	-	-	-	-	-	-	-	-	0.80	0.62–0.92	0.077
36	2023	Wei et al. [62]	-	-	-	-	0.77	0.88	-	-	0.87	0.87–0.88	0.003
37	2023	Wen et al. [63]	-	-	-	-	0.89	0.70	-	-	0.85	0.77–0.93	0.041
38	2023	Peng W. et al. [64]	-	-	-	-	-	-	-	-	0.80	0.6-1	0.102
39	2023	Peng J. et al. [65]	-	-	-	-	0.86	0.89	-	-	0.94	0.87–0.98	0.028
40	2023	Ouyang et al. [66]	-	-	-	-	0.93	0.86	0.88	0.92	0.87	-	-
41	2023	Miranda et al. [67]	-	-	-	-	0.85	0.67	0.36	0.95	0.76	0.69–0.83	0.036
42	2023	Ke et al. [68]	53	11	4	167	0.93	0.94	0.83	0.98	0.95	0.92–0.98	0.015
43	2023	Huang et al. [69]	-	-	-	-	0.84	0.81	0.57	0.95	0.87	0.78–0.95	0.043
44	2024	Homsi et al. [70]	-	-	-	-	1	0.81	-	-	0.9	0.67-1	0.084

(*Standard error (SE) calculated from AUC 95% confidence interval)

**Table 4:** Subgroup analysis

Subgroup	Number of studies	I^2^%	Pooled AUC Random effects		95% CI	SE	*P* value*
**MRI Timing**							
Pre-nCRT	19	93.86		0.86	0.821–0.897	0.019	0.43
Pre-nCRT & Post-nCRT	12	93.47		0.88	0.833–0.933	0.0256	
**MRI field intensity**							
1.5 T	6	1.96		0.82	0.797–0.833	0.009	**< 0.001**
3 T	13	93.11		0.90	0.865–0.940	0.019	
**Radiomic feature**							
≤ 1000	11	95.16		0.86	0.810–0.907	0.025	0.86
> 1000	14	88.03		0.86	0.821–0.907	0.022	
**Classification**							
Traditional radiomics	27	97.93		0.86	0.829–0.889	0.015	0.48
Deep learning	8	86.90		0.89	0.819–0.951	0.034	
**Model**							
Radiomics	21	89.52		0.86	0.823–0.893	0.018	0.56
Combined	14	98.73		0.88	0.830–0.921	0.023	
**Radiomics Quality Score**							
< 12	14	77.59		0.85	0.820–0.886	0.017	0.48
> 12	16	98.59		0.87	0.827–0.921	0.024	
**Ethnicity**							
Asian	22	98.26		0.90	0.870–0.927	0.015	**< 0.001**
Non-Asian	12	73.77		0.80	0.749–0.844	0.024	

(*Handley & McNeil test for statistical significance of the difference between the AUC of independent ROC curves)

### Radiomics model

The most common software used to segment the region of interest was ITK-SNAP [71]. Three studies (7%) used fully automated segmentation of regions of interest while three studies reported using semiautomatic and the majority of studies used manual segmentation. PyRadiomics software [72] was used in seventeen studies (39%) for radiomics features extraction being the most common. Radiomic features were extracted from pre-treatment MRI only in twenty-seven studies (61%), thirteen studies (30%) used pre- and post-treatment imaging. Three studies (7%) used post-treatment MRI only. T2-weighted imaging was used in forty-three studies (98%) and one study used diffusion-weighted imaging. Thirty-seven studies (84%) have reported internal validation. Eighteen studies (41%) have reported external validation. See Table 2 for MRI-derived radiomics model characteristics.

### Quality assessment

**Fig. 3:**
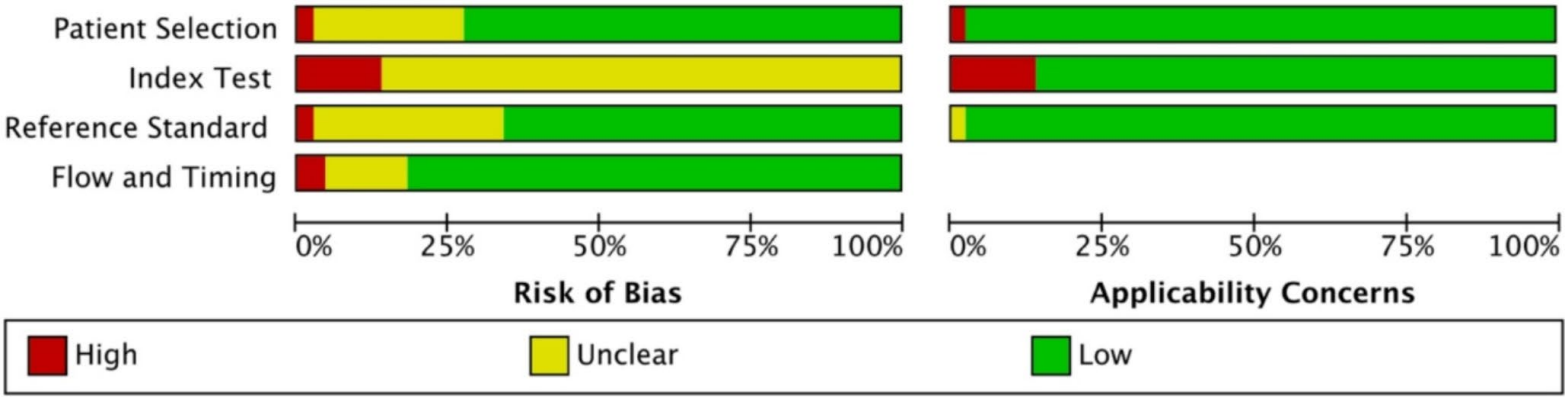
Quality assessment of diagnostic accuracy studies (QUADAS-2)

The methodological quality of QUADAS-2 is displayed in Fig. 3. The risk of bias was high or unclear in twelve studies (27%) for patient selection. All the studies were noted to have high or unclear risk of bias for index test. Fifteen studies (34%) were at high or unclear risk of bias for reference standard. The risk of bias was high or unclear in eight studies (18%) for flow and timing. In terms of applicability of concerns one study was noted to have high concern of patient selection. Six studies (14%) were found to have high concern for index text. There was one study noted to have unclear concern from reference standard.

**Fig. 4:**
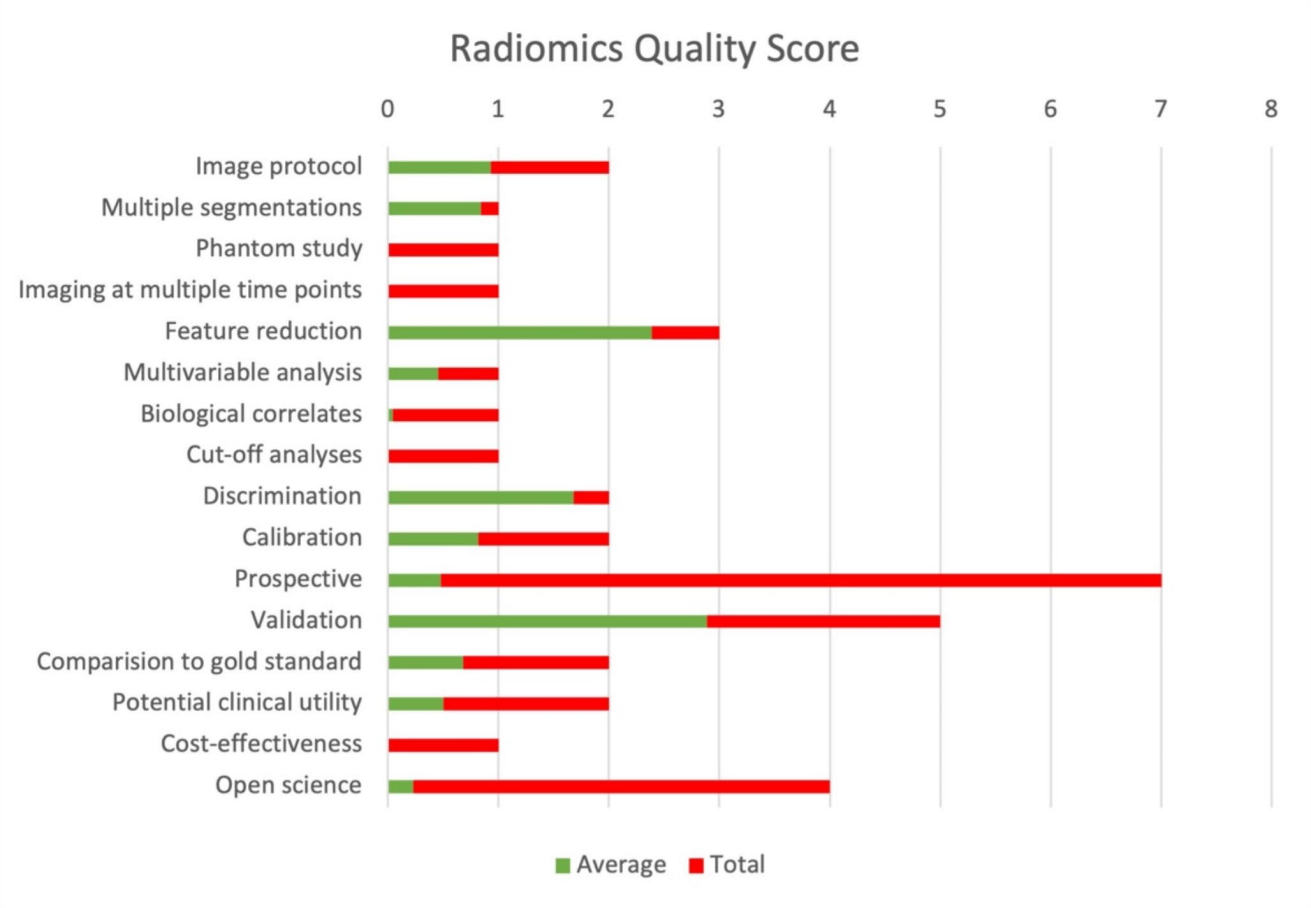
Radiomics quality score (RQS)

The average RQS was 12 (range 4–24). The average score for each category is displayed in Fig. 4. There was no study considering “phantom study”, “imaging at multiple time points”, “cut-off analyses” and “cost-effectiveness” leading to score of zero in these categories. There was no significant publication bias (Egger’s intercept − 1.68, CI -4.15 to 0.78, *p* = 0.17).

### Diagnostic accuracy

**Fig. 5:**
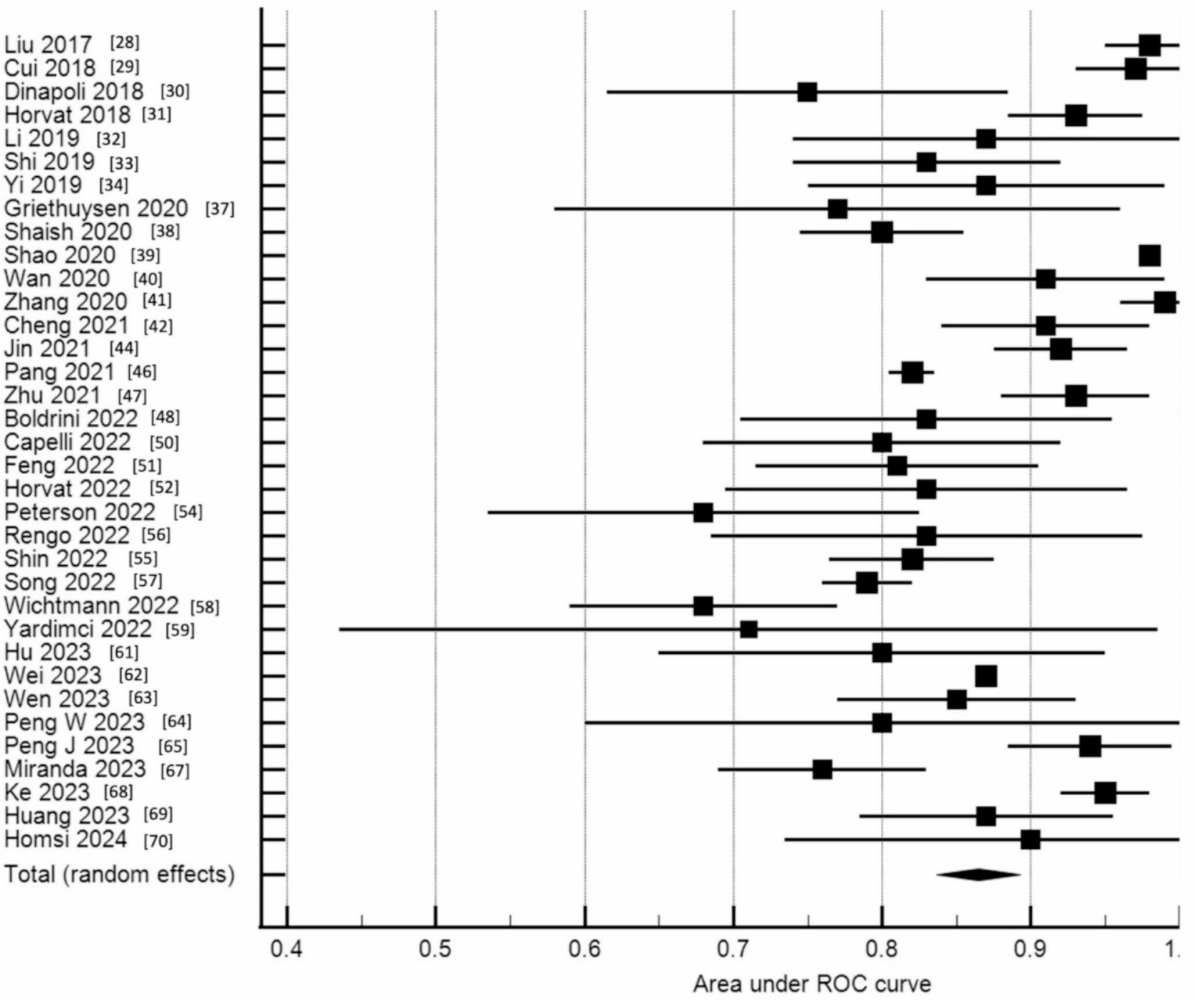
Forest plot of pooled area under the ROC curve

Table 3 shows the summary of performance of the MRI derived radiomics models. Twelve studies (27%) demonstrated excellent discrimination with AUC > 0.9. Eighteen studies (41%) had good discrimination with AUC between 0.81 and 0.9. Twelve studies (27%) reported moderate discrimination with AUC between 0.71 and 0.8. Two studies (5%) reported poor discrimination with AUC < 0.7. In the meta-analysis including thirty-five studies the pooled AUC was 0.87 (random-effects model) with 95% CI 0.84–0.89. See Fig. 5 for forest plot of pooled AUC included in the meta-analysis. Nine studies (20%) were excluded from the meta-analysis as these studies did not report on 95% CI for AUC so the standard error could not be calculated. The study identified from additional record using manual search was not selected for the meta-analysis due to this same reason. There was high heterogeneity between studies with I^2^ = 97.4% (*p* < 0.001).

### Subgroup analysis

Subgroup analysis was performed on the thirty-five studies included in the meta-analysis. To allow comparison of two independent ROC curves the method described by Hanley & McNeil was used [73]. This method uses the AUC and standard error for the two ROC curves and p-value < 0.05 is considered significantly different. In the sub-group analysis, the 3 T MRI field intensity had a superior pooled AUC of 0.9 compared to pooled AUC of 0.8 for 1.5 T (*p* < 0.001). The pooled AUC of Asian ethnicity was 0.9 compared to non-Asians with pooled AUC of 0.8 (*p* < 0.001). See Table 4 for results of the subgroup analysis. There was no significant difference in subgroup analysis when comparing MRI timing, number of radiomic features extracted, classification method, combined model and radiomics quality score.

## Discussion

In this systematic review we have assessed forty-four MRI radiomics prediction models for pCR following neoadjuvant treatment in LARC. Most studies had small sample sizes and lacked external validation. Despite these limitations, in our meta-analysis that included thirty-five studies, the MRI radiomics models demonstrate accurate discriminatory performance with a pooled AUC of 0.87. Jia et al. reported a meta-analysis including twenty-one studies evaluating the diagnostic accuracy of artificial intelligence (AI) models with MRI for predicting pCR to nCRT in rectal cancer patients [74]. In their study the pooled AUC was reported to be 0.91 (95% CI 0.88–0.93) with pooled sensitivity 0.82 and specificity 0.86 demonstrating the highly predictive value using MRI-derived radiomics models. Furthermore, subgroup analysis showed deep learning (DL) models had higher predictive accuracy than traditional radiomics model, and combined radiomics models incorporating clinical factors had higher diagnostic accuracy than using radiomics models alone.

Traditional radiomics models involve extraction of human-defined quantitative features which describe tumour shape and texture, among other characteristics. The final step uses machine learning (ML) methods to identify radiomics features associated with a given clinical endpoint [75]. In contrast, DL methods allow automated learning of relevant radiomic features without the need for previous definition by the researchers [75]. The human derived nature of traditional radiomics methods could introduce human bias into the process which may be minimised by the DL methods.

In our study we were not able to demonstrate the superiority of combined radiomics models. In contrast to the previous study, the use of DL radiomics models did not outperform traditional radiomics models in our subgroup analysis [74]. These findings may be explained by the larger number of studies included in our meta-analysis and the different methodology applied for subgroup analysis. Interestingly, we have compared independent pooled AUC and demonstrated superior performance with 3 T MRI system. The 3 T field strength has increased signal-to-noise ratio, spatial resolution, temporal resolution, specific absorption rate and acoustic noise. Ammari et al. showed that field strength influences numerous texture features values, spatial resolution and gray scale resampling in brain MRI suggesting the radiomics features at 1.5 T and 3 T are not interchangeable [76].

We have compared pooled AUC values of studies with Asian and non-Asian ethnicity. The twenty-two Asian studies included populations from China and Korea. The twelve western studies included populations from Italy, USA, Brazil, Netherlands, Germany and Turkey. The predictive performance for Asian ethnicity was significantly higher than non-Asian ethnicity with pooled AUC of 0.9 and 0.8, respectively. Dercle et al. has reported the difference in CT-derived radiomics phenotype between different ethnicities after systemic treatment for metastatic colorectal cancer [77]. CT radiomics features were associated with poor overall survival among African Americans with pancreatic ductal adenocarcinoma when compared to non-African Americans [78]. To our knowledge ethnicity has not yet been reported to have an influence on MRI radiomics features in LARC. Future studies will need to explore this relationship between ethnicity and MRI radiomics.

Radiomics research in rectal cancer has been reported in tumour stage classification, lymph node metastasis, liver metastasis, KRAS mutation and MSS status [79–84]. Ma et al. reported a high resolution T2WI MRI-based radiomics model for differentiating tumour stage and nodal stage using the SVM classification with AUC of 0.86 (95% CI 0.75–0.97) [79]. Lin et al. demonstrated that higher MRI radiomics score predicted locally advanced rectal cancer independent of tumour diameter, location, CEA and CA 19 − 9 in a multivariate analysis [80]. A systematic review and meta-analysis reported the radiomics models in colorectal cancer outperformed the radiologist in predicting lymph node metastasis with pooled AUC of 0.73 (95% CI 0.63–0.82) and 0.68 (95% CI 0.63–0.73) respectively [81]. Liang et al. have reported AUC of 0.87 when investigating the performance of MRI radiomics for prediction of metachronous liver metastasis in rectal cancer [82]. Cui et al. developed MRI radiomics prediction of KRAS mutation in rectal cancer with moderate performance reporting AUC of 0.71 in external validation [83]. These studies demonstrate the potential application of radiomics in enhancing rectal cancer staging and improving risk prediction.

We have assessed methodical quality using QUADAS-2 and reported the risk of bias was high or unclear for patient selection in twelve studies as there was insufficient detail on the consecutive or random sampling of patients enrolled. This could have introduced selection bias. To mitigate this risk, future studies will need to report the patient selection process including the reasons for excluding patients. All the included studies were noted to have high or unclear risk of bias for index test as there is no reference standard or pre-specified threshold in the radiomics study; this may overestimate the diagnostic performance. A recent study exploring publication bias of radiomics studies has concluded that there is strong bias towards publishing positive results [85]. To advance the field of radiomics research both positive and negative results must be published.

Furthermore, the field of radiomics currently lacks standardisation. Collaborations between researchers and clinicians are important to develop standards for MRI acquisition and analysis. Another important aspect is the utilisation of data and data sharing techniques. In the future the extraction of radiomics features could be stored and shared in a large database network across various departments and institutions. This vast accumulation of large datasets would enhance radiomics research by improving standardisation, model development and validation.

The average RQS in our study was twelve compared to eleven in the previous study [74]. The subgroup analysis in our study comparing low and high RQS did not reach statistical significance. Most studies were retrospective and opportunistic using the pre-treatment MRI as part of local staging. None of the studies collected MRI images of individuals at additional time points to assess for temporal variabilities such as tissue movement, organ expansion or shrinkage. Studies that have assessed for delta radiomics, which is the analysis of variation in radiomics features between pre- and post-nCRT, we gave a score of zero for “imaging at multiple time points” as nCRT would have altered the tumour and extracted radiomics features rather than capture the true temporal variability. None of the radiomics studies have used previously published cut-off(s), nor performed cost-effective analysis. These were the reasons for most studies missing scores, whilst studies that used prospective validation scored above the average RQS.

Our study does have several limitations. There is heterogeneity between different studies, so we performed subgroup analysis to explore potential sources. We have identified differences related to MRI field intensity and ethnicity based on Asian and non-Asian. The majority of the studies were representative of a Chinese population and the widespread generalisability of these results will be limited. Despite these attempts we will not be able to identify all sources of heterogeneity.

It is likely that our study has publication bias due to the lack of negative results being published in radiomics research and this may overestimate the predictive performance for pCR. Many studies identified in our systematic review did not report the values of true positive, false positive, true negative and false negative to derive pooled sensitivity and specificity for diagnostic test accuracy. To maximise the number of studies included for the meta-analysis we have performed pooled AUC analysis using reported AUC and standard error calculated from 95% CI. This method has been applied in previous published meta-analysis [81]. However, we were not able to perform complex diagnostic meta-analysis or hierarchical summary receiver operating characteristic analysis. Again, due to this limitation we have not performed meta-regression analysis. To explore the source for high heterogeneity we have performed subgroup analysis.

## Conclusion

The current state of MRI-derived radiomics studies mostly lack external validation and large multicentre prospective studies are limited. We have demonstrated that 3 T MRI field provides a superior predictive performance, whilst the role of ethnicity on radiomics features needs to be explored in future studies. Further research in the field of MRI radiomics is important as accurate prediction for pCR can lead to an organ preservation strategy in LARC.

## Electronic supplementary material

Below is the link to the electronic supplementary material.

Supplementary Material 1

## Data Availability

No datasets were generated or analysed during the current study.
