# Supplementary material for: MRI radiomics prediction modelling for pathological complete response to neoadjuvant chemoradiotherapy in locally advanced rectal cancer: a systematic review and meta-analysis
Source: Abdom Radiol (NY). 2025 Apr 28;50(11):5103–23. doi: 10.1007/s00261-025-04953-5 (PMC12568919; doi:10.1007/s00261-025-04953-5)

**Table 1: Result of search strategy**

| **Databases searched** | **Date of search** | **Number of results** |
| --- | --- | --- |
| **Ovid MEDLINE(R) ALL <1946 to February 01, 2024>** | 02/02/2024 | 326 |
| **Embase <1974 to 2024 February 01>** | 02/02/2024 | 582 |
| **Cochrane Central Register of Controlled Trials (CENTRAL) and Cochrane** | 02/02/2024 | 31 |
| **Total results after deduplication** | **610 (after subtracting 329 duplicates)** | |

**Table 2: Search result from Ovid MEDLINE**

| **Database:** | **Ovid MEDLINE(R) ALL <1946 to February 01, 2024>** | **Results per line:** | **Number of results:** |
| --- | --- | --- | --- |
| **Date:** | **02/02/2024** |  |  |
| 1 | Rectal Neoplasms/ | 49182 | **326** |
| 2 | ((rectal* or rectum*) adj3 (neoplas* or cancer* or tumo?r* or malignan* or adenocarcinoma* or mass*)).ti,ab,kw,kf. | 43462 |  |
| 3 | 1 or 2 | 63627 |  |
| 4 | exp Magnetic Resonance Imaging/ | 543023 |  |
| 5 | ((magnetic resonance or magnetic-resonance or diffusion magnetic or diffusion-magnetic or diffusion tensor or diffusion-tensor or diffusion weighted or diffusion-weighted or echo planar or echo-planar or echo magnetic or echo-magnetic or contrast enhanced or contrast-enhanced or T1 weighted or T1-weighted or T2 weighted or T2-weighted or "fast-spin echo" or "rapid-spin echo" or spin echo or spin-echo or nuclear magnetic or nuclear-magnetic or "MR" or "MR-" or "MRI" or "MRI-" or "-MRI" or "MRA" or "MRA-" or "DW" or "DW-" or "DWI" or "DWI-" or "EPI" or "EPI-" or "HASTE" or "HASTE-" or "NMR" or "NMR-") adj8 (imag* or scan* or screen* or detect* or diagnos* or sialogra* or tractograph* or neurogra* or venograph* or angiogra* or perfusion* or tomograph*)).ti,ab,kw,kf. | 530394 |  |
| 6 | 4 or 5 | 759036 |  |
| 7 | exp Artificial Intelligence/ | 188937 |  |
| 8 | (radiomic* or AI or ((machine* or deep*) adj1 learning*) or ((artificial* or machine* or computational*) adj1 intelligence*) or neural network* or neural-network* or automatic detection* or support vector* or computer reasoning*).ti,ab,kw,kf. | 299924 |  |
| 9 | 7 or 8 | 375497 |  |
| 10 | 3 and 6 and 9 | 326 |  |
| 11 | 20240201.up. | 20727 |  |
| 12 | 10 not 11 | 326 |  |

**Table 3: Search result from Embase**

| **Database:** | **Embase <1974 to YYYY Week 00>** | **Results per line:** | **Number of results:** |
| --- | --- | --- | --- |
| **Date:** | **DD/MM/YYYY** |  |  |
| 1 | exp rectum cancer/ | 59374 | **582** |
| 2 | ((rectal* or rectum*) adj3 (neoplas* or cancer* or tumor* or malignan* or adenocarcinoma* or mass*)).ti,ab,kw,kf. | 63192 |  |
| 3 | 1 or 2 | 80921 |  |
| 4 | exp nuclear magnetic resonance imaging/ | 1280149 |  |
| 5 | ((magnetic resonance or magnetic-resonance or diffusion magnetic or diffusion-magnetic or diffusion tensor or diffusion-tensor or diffusion weighted or diffusion-weighted or echo planar or echo-planar or echo magnetic or echo-magnetic or contrast enhanced or contrast-enhanced or T1 weighted or T1-weighted or T2 weighted or T2-weighted or "fast-spin echo" or "rapid-spin echo" or spin echo or spin-echo or nuclear magnetic or nuclear-magnetic or "MR" or "MR-" or "MRI" or "MRI-" or "-MRI" or "MRA" or "MRA-" or "DW" or "DW-" or "DWI" or "DWI-" or "EPI" or "EPI-" or "HASTE" or "HASTE-" or "NMR" or "NMR-") adj8 (imag* or scan* or screen* or detect* or diagnos* or sialogra* or tractograph* or neurogra* or venograph* or angiogra* or perfusion* or tomograph*)).ti,ab,kw,kf. | 746671 |  |
| 6 | 4 or 5 | 1403428 |  |
| 7 | radiomics/ | 10080 |  |
| 8 | exp artificial intelligence/ | 95341 |  |
| 9 | exp machine learning/ | 450056 |  |
| 10 | (radiomic* or "AI" or ((machine* or bayesian* or deep*) adj1 learning*) or ((artificial* or machine* or computational*) adj1 intelligence*) or neural network* or neural-network* or automatic detection* or support vector* or computer reasoning*).ti,ab,kw,kf. | 362201 |  |
| 11 | 7 or 8 or 9 or 10 | 603585 |  |
| 12 | 3 and 6 and 11 | 583 |  |
| 13 | 20240201.dd. | 6359 |  |
| 14 | 12 not 13 | 582 |  |

**Table 4: Search result from Cochrane Central Register of Controlled Trials**

| **Database:** | **Cochrane Central Register of Controlled Trials (CENTRAL)** | **Results per line:** | **Number of results:** |
| --- | --- | --- | --- |
| **Date:** | **02/02/2024** |  |  |
| #1 | MeSH descriptor: [Rectal Neoplasms] this term only | 2650 | **31** |
| #2 | ((rectal* or rectum*) NEAR/3 (neoplas* or cancer* or tumo?r* or malignan* or adenocarcinoma* or mass*)):ti,ab,kw | 5722 |  |
| #3 | #1 or #2 | 5722 |  |
| #4 | MeSH descriptor: [Magnetic Resonance Imaging] explode all trees | 12810 |  |
| #5 | ((magnetic NEXT resonance OR echo NEXT magnetic OR echo NEXT planar OR "fast spin echo" OR diffusion NEXT tensor OR T1 NEXT weighted OR T2 NEXT weighted OR MR OR MRI OR MRA OR DW OR DWI OR EPI OR NMR) NEAR/5 (imag* or scan* or screen* or detect* or diagnos* or sialogra* or tractograph* or neurogra* or venograph* or angiogra* or perfusion* or tomograph*)):ti,ab,kw | 39863 |  |
| #6 | #4 or #5 | 39881 |  |
| #7 | MeSH descriptor: [Artificial Intelligence] explode all trees | 3108 |  |
| #8 | (radiomic* or AI or ((machine* or bayesian* or deep*) NEXT learning*) or ((artificial* or machine* or computational*) NEXT intelligence*) or neural NEXT network* or automatic NEXT detection* or support NEXT vector* or computer NEXT reasoning*):ti,ab,kw | 11598 |  |
| #9 | #7 or #8 | 12948 |  |
| #10 | #3 and #6 and #9 | 31 |  |
| #11 | #10 in Trials | 31 |  |

**Table 5: Radiomics Quality Score**

| **Points** | **Deduct** | **Description** |
| --- | --- | --- |
| 2 | 0 | Image protocol - well documented, contrast, slice thickness, energy |
| 1 | 0 | Multiple segmentations - by different physicians/algorithm/software |
| 1 | 0 | Phantom study on all scanners - detect inter scanner differences and vendor dependent features |
| 1 | 0 | Imaging at multiple time points |
| 3 | 3 | Feature reduction or adjustment for multiple testing - decrease overfitting. |
| 1 | 0 | Multivariable analysis with non radiomics features |
| 1 | 0 | Detect and discuss biological correlates |
| 1 | 0 | Cut off analysis - using median, published cut off or report continous |
| 2 | 0 | Discrimination statistics - C-statistic, ROC curve, AUC (p-value, CI) |
| 2 | 0 | Calibration statistics - Bootstrapping, cross validation |
| 7 | 0 | Prospective study - supports clinical validity and usefulness |
| 5 | 5 | Validation - performed without retraining and without adaptation of cut off value |
| 2 | 0 | Comparison to gold standard - added value of radiomics |
| 2 | 0 | Potential clinical utility |
| 1 | 0 | Cost effectiveness analysis - report on the cost effectiveness of the clinical application |
| 4 | 0 | Open science and data - make code and data publicly available |

**Figure 1: Assessment of risk of bias of studies using QUADAS-2 tool**

**
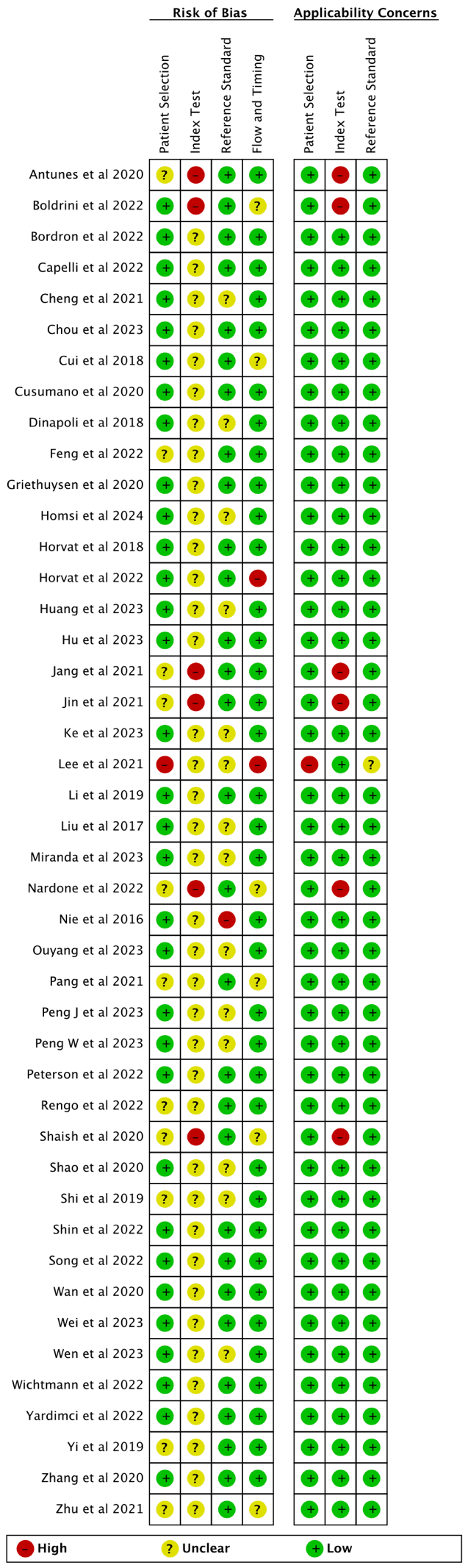
**

**Figure 2: Funnel plot**


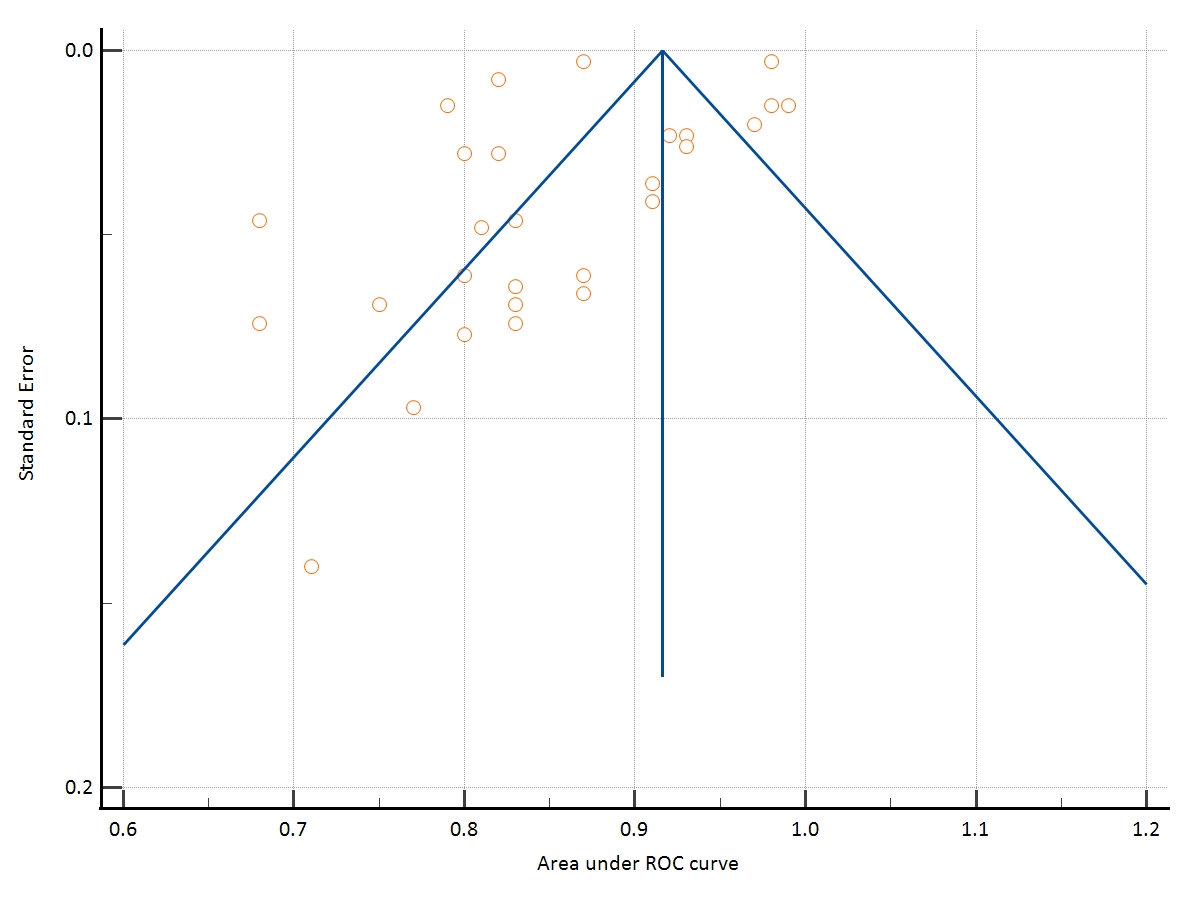

Supplement: Supplementary file 1 — Supplementary Material 1 [file 261_2025_4953_MOESM1_ESM.docx]
